# Can More Nanoparticles Induce Larger Viscosities of Nanoparticle-Enhanced Wormlike Micellar System (NEWMS)?

**DOI:** 10.3390/ma10091096

**Published:** 2017-09-18

**Authors:** Mingwei Zhao, Yue Zhang, Chenwei Zou, Caili Dai, Mingwei Gao, Yuyang Li, Wenjiao Lv, Jianfeng Jiang, Yining Wu

**Affiliations:** State Key Laboratory of Heavy Oil Processing, School of Petroleum Engineering, China University of Petroleum (East China), Qingdao 266580, China; zhaomingwei@upc.edu.cn (M.Z.); upczhangyue@163.com (Y.Z.); zoucwupc@163.com (C.Z.); gaomingwei93@163.com (M.G.); sunliyuyang@163.com (Y.L.); lwj940502@163.com (W.L.); jjf19950926@126.com (J.J.)

**Keywords:** nanoparticle, viscosity, NEWMS, viscoelasticity, temperature

## Abstract

There have been many reports about the thickening ability of nanoparticles on the wormlike micelles in the recent years. Through the addition of nanoparticles, the viscosity of wormlike micelles can be increased. There still exists a doubt: can viscosity be increased further by adding more nanoparticles? To answer this issue, in this work, the effects of silica nanoparticles and temperature on the nanoparticles-enhanced wormlike micellar system (NEWMS) were studied. The typical wormlike micelles (wormlike micelles) are prepared by 50 mM cetyltrimethyl ammonium bromide (CTAB) and 60 mM sodium salicylate (NaSal). The rheological results show the increase of viscoelasticity in NEWMS by adding nanoparticles, with the increase of zero-shear viscosity and relaxation time. However, with the further increase of nanoparticles, an interesting phenomenon appears. The zero-shear viscosity and relaxation time reach the maximum and begin to decrease. The results show a slight increasing trend for the contour length of wormlike micelles by adding nanoparticles, while no obvious effect on the entanglement and mesh size. In addition, with the increase of temperature, remarkable reduction of contour length and relaxation time can be observed from the calculation. NEWMS constantly retain better viscoelasticity compared with conventional wormlike micelles without silica nanoparticles. According to the Arrhenius equation, the activation energy *E_a_* shows the same increase trend of NEWMS. Finally, a mechanism is proposed to explain this interesting phenomenon.

## 1. Introduction

Since 1980’s nanocrystals have been firstly prepared by manual work, research studies and applications of nanostructured materials have received widespread attention [1,2]. Nanoparticles often refer to very small solid particles, which range from 1 to 100 nm [3]. Due to the small size, nanoparticles show special properties, such as large specific surface area and adsorption performance [4,5,6,7,8]. The study of materials modification by adding nanoparticles has been developed in the recent years, where enhancing the strength of wormlike micelles by adding nanoparticles is one of the focuses.

Recently, viscoelastic surfactants have attracted more researchers’ attention due to good viscoelasticity, sand suspension, stability and environmentally friendliness [9]. Under different concentrations, viscoelastic surfactant molecules can form different aggregates, just like rodlike micelles, wormlike micelles, vesicles, lamellar phases and liquid crystals, in which wormlike micelles are used widely in fracturing fluid due to better viscoelasticity [10,11,12]. Viscosity of conventional wormlike micelles can decrease greatly with the increase of temperature, which seriously results in instability limitation in complex environments [13,14]. As previously mentioned, in order to enhance the strength of wormlike micelles, some researchers have done a large amount of work, such like adding silica and titania nanoparticles for enhancing wormlike micelles [15,16,17]. Due to the tiny size and high specific area, adding nanoparticles in wormlike micelles can affect molecular interaction between each other [18,19]. It is expected that special properties of nanoparticles can enhance structure strength of micelles by microcosmic function, which can be reflected in the resistance of viscosity at increased temperature [16]. Nettesheim et al. reported the viscoelasticity of wormlike micelles by cetyltrimethyl ammonium bromide (CTAB) and sodium nitrate (NaNO_3_) with the addition of silica nanoparticles and found that the zero-shear viscosity (*η*_0_) and relaxation time (*τ_R_*) of solutions would be increased by adding a small amount of silica nanoparticles [20]. In addition, with the decrease of CTAB concentration, tackifying effects of the same amount of nanoparticles added in wormlike micelles are more remarkable than before. Helgeson et al. did further study on the tackification mechanism of wormlike micelles by silica nanoparticles [21]. They proposed a newly micelle-particle structure existing in CTAB/NaNO_3_ wormlike micellar solution with addition of silica nanoparticles, which can be called “Double Networks”. The observation by cryogenic transmission electron microscopy (cryo-TEM) was used to verify this hypothesis. Luo et al. used the anionic surfactant fatty acid methyl ester sulfonate sodium to prepare wormlike micelles with the aid of barium titanate (BaTiO_3_) nanoparticles [16]. Their work investigated influence of different factors on the viscoelasticity of wormlike micelles, such as surfactant concentration, mass fraction of nanoparticles and temperature.

Until now, most of these research studies indicate that with the increase of nanoparticle concentrations, viscosities of wormlike micelles increase. If further adding nanoparticles, can more nanoparticles induce larger viscosities of nanoparticle-enhanced wormlike micellar system (NEWMS)? In addition, the effect of temperature on nanoparticle-enhanced wormlike micelles has been not studied sufficiently. Therefore, it is necessary to carry out the related research.

In this work, the effects of nanoparticle concentration and temperature on the NEWMS were studied. The conventional wormlike micelle is formed by CTAB and sodium salicylate (NaSal), which is one of the most widely applied formula at present. NEWMS were prepared by 50 mM CTAB and 60 mM NaSal with the addition of silica nanoparticles. Rheological measurements were conducted to evaluate the rheological properties of NEWMS. It is observed that with the addition of silica nanoparticles, the viscosity of wormlike micelles is increased, demonstrating that nanoparticles induce micellar growth. However, with the increase of nanoparticle concentration, the viscosity of NEWMS is decreased but still higher than that of conventional wormlike micelles. In addition, effects of nanoparticles and temperature on the entanglement length, mesh size and contour length of wormlike micelles are clarified. For explaining this phenomenon, a new mechanism of crosslinking between nanoparticles and wormlike micelles is proposed.

## 2. Materials and Methods

### 2.1. Materials

The CTAB and NaSal were purchased from Shanghai Experimental Reagent Co., Ltd. (Shanghai, China) without further purification. Hydrophilic silica nanoparticles with the average of 60 nm were supplied by Molinhui New Advanced Materials Co., Ltd. (Shanghai, China). Water was triply-distilled.

### 2.2. Sample Preparation

Silica nanofluids are prepared by distilled water at different mass fractions, including 0.1%, 0.3%, and 0.5%. Firstly, samples are mixed by mechanical stirrer for 1 h, and then separated by ultrasonic dispersion for 4 h at 60 °C. The model of ultrasonic dispersion device was KQ3200DE, supplied by Kunshan Ultrasonic Instruments Co., Ltd (Kunshan, China). Then, the nanofluid is regarded as the base fluid, which is used to prepare CTAB solution (100 mM) and NaSal solution (120 mM). Then, CTAB solution and NaSal solution are mixed together in equal volume at 50 °C. After mixing for 1 h, NEWMS were prepared. In addition, wormlike micelle without silica nanoparticles was regarded as a contrast sample.

### 2.3. Rheological Measurements

The rheological properties of samples were measured by using Haake Mars 60 rheometer (Thermo Fisher Scientific, Karlsruhe, Germany) with the cone plate system (diameter 35 mm; angle 1°). The range of shear rate is kept from 0.01 to 300 s^−1^ during the steady shear measurement. In oscillatory measurements, the frequency was kept at 6.28 rad·s^−1^ (1 Hz) with the variation of the stress (σ). When the linear viscoelastic region was confirmed, frequency sweep measurements were performed as a function of frequency at a constant stress. The temperature of measurements was regarded as the control variable in this work. These wormlike micelles samples were tested at different temperatures, such as 20, 30, and 40 °C.

## 3. Results and Discussion

### 3.1. Rheological Properties of NEWMS

**Figure 1:**
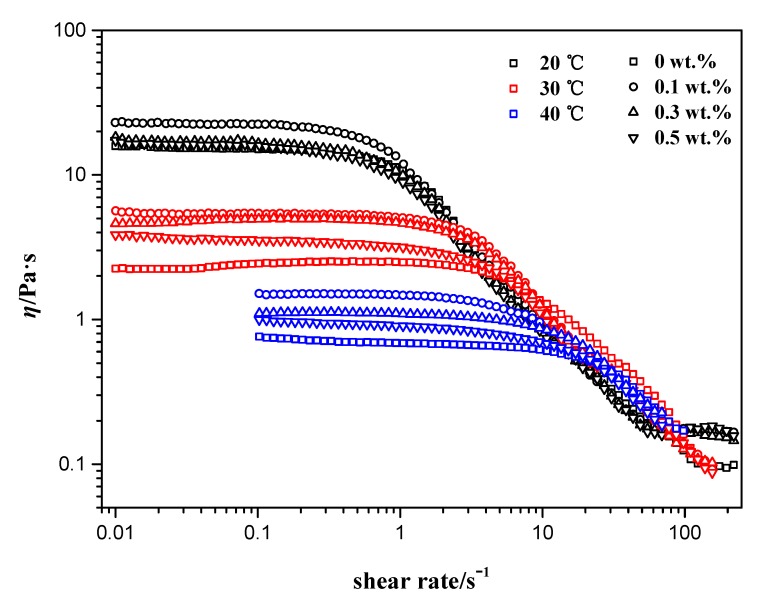
Steady shear viscosities of NEWMS with addition of different silica mass fraction at 20, 30, and 40 °C.

In order to investigate the influence of added nanoparticles on NEWMS at elevated temperatures, the steady shear measurements are conducted firstly. Figure 1 shows the different shear-rate viscosities of NEWMS with different silica concentrations at 20, 30, and 40 °C. It can be observed that all viscosities keep constant at low shear rate, and this plateau value of shear viscosity can be regarded as the zero-shear viscosity (*η*_0_), which is the significant assessment factor of rheological property. From Figure 1, it is clear that the addition of silica nanoparticles can promote the viscosity of wormlike micelles.

As can be seen from Figure 1, with the further increase of shear rate, viscosities become smaller and show remarkable shear thinning phenomenon, which is the representative symbol of wormlike micelles formation [12,22,23,24]. The high shear rates lead to the alignment of aggregates in micelles, and make shear banding phenomenon eventually. At low shear rates, NEWMS with 0.1 wt % nanoparticles has the highest zero-shear viscosity (*η*_0_) through comparison. This phenomenon illustrates that the addition of silica nanoparticles can indeed improve the viscosity of NEWMS. However, with further increase of nanoparticles, the zero-shear viscosity begins to decrease.

**Figure 2:**
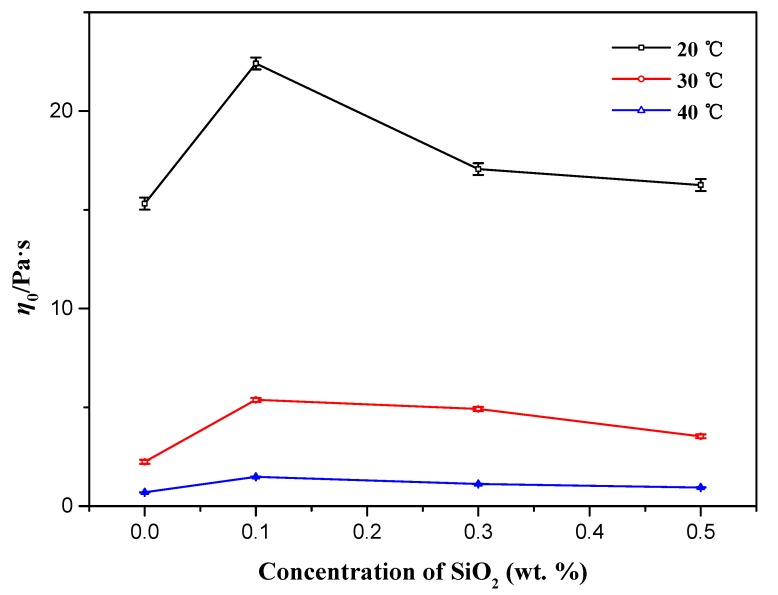
Zero-shear viscosity (*η*_0_) of NEWMS with different nanoparticle concentrations at 20, 30, and 40 °C.

From Figure 2, it can be seen clearly that with the increase of temperature, steady shear viscosities become smaller dramatically, which can be interpreted as the acceleration of the dynamic process of breaking and recombination of micelles with temperature increasing [25]. Similarly, even at different temperatures, NEWMS still have larger viscosities than wormlike micelles without nanoparticles. The NEWMS with the addition of 0.1 wt % silica nanoparticles still retain the highest *η*_0_ at different temperatures.

**Figure 3:**
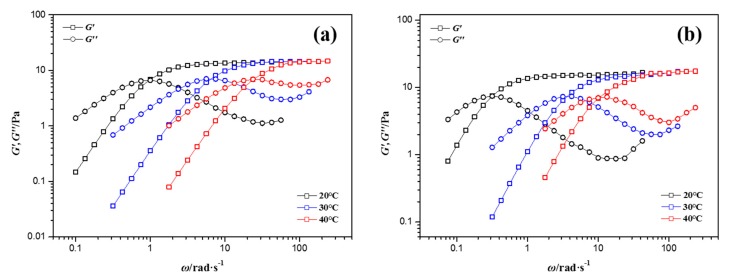

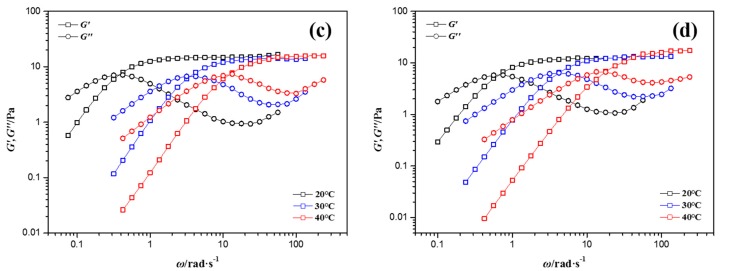
Linear viscoelastic spectrum of NEWMS at different temperatures as a function of silica nanoparticle mass fractions (**a**) 0 wt % silica; (**b**) 0.1 wt % silica; (**c**) 0.3 wt % silica; and (**d**) 0.5 wt % silica.

To further study the effects of nanoparticle concentrations and elevated temperatures on micellar viscoelasticity, dynamic oscillatory measurements were conducted. As can be seen from Figure 3, the storage modulus *G′* and loss modulus *G″* vary with oscillation frequencies, and all NEWMS exhibit typical features of wormlike micelles at elevated temperatures. *G′* and *G″* increase with the increase of frequency. At low shear frequencies, *G″* is larger than *G′*, showing that NEWMS have more viscous properties, while at high shear frequencies, *G′* is larger than *G″*, showing to be more elastic. It can be observed that *G′* reaches a constant value, which is the plateau modulus *G*_0_, and *G″* can reach the minimum value, which is determined as *G″*_min_.

For typical wormlike micelles, a simple Maxwell model is generally used to investigate rheological properties [26,27]. As to the Maxwell fluid, *G′* and *G″* can be calculated according to Equations (1) and (2) [22]:

(1) $$G^{\prime }=\frac{G_{0}\omega^{2}\tau_{R}^{2}}{1+\omega^{2}\tau_{R}^{2}}$$

(2) $$G^{\prime \prime }=\frac{G_{0}\omega \tau_{R}}{1+\omega^{2}\tau_{R}^{2}}$$

In these equations, *ω* is the angular frequency and *τ_R_* is the micelle relaxation time. The relaxation time *τ_R_* is an significant factor for estimating rheological properties of wormlike micelles, which can be calculated according to Equation (3) proposed by Cates [22]:

(3) $$\tau_{R}=\frac{1}{\omega_{co}}$$

where *ω_co_* is the angular frequency of crossover point while storage modulus *G′* is the same as the value of loss modulus *G″*.

The Cole-Cole plot is usually used to evaluate whether the data of *G′* and *G″* fit the Maxwell model well [12,28]. As for this work, Cole-Cole plots (a curve of *G″* as a function of *G′*) are studied from the following Equation (4) [22]:

(4) $$G^{\prime \prime }+{\left(G^{\prime }-\frac{G_{0}}{2}\right)}^{2}={\left(\frac{G_{0}}{2}\right)}^{2}$$

**Figure 4:**
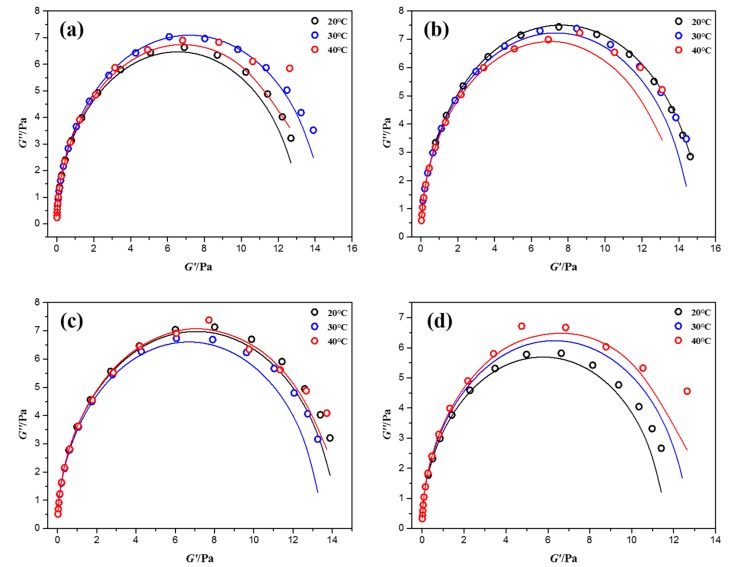
Cole-Cole plots for NEWMS at different temperatures as a function of the silica nanoparticle mass fraction (**a**) 0 wt % silica; (**b**) 0.1 wt % silica; (**c**) 0.3 wt % silica; and (**d**) 0.5 wt % silica.

Figure 4 shows the plots of *G″* versus *G′* of NEWMS. It is observed that Cole-Cole plots of these NEWMS fit well with the calculated results at low shear frequencies. While at high shear frequencies, practical data begin to deviate from theoretical semicircle in the Cole-Cole plot. This phenomenon can be explained by the appearance of Rouse modes or “breather modes” [14,26], which is usually observed in other wormlike micelles reported before [16,25].

To further investigate the rheological properties of NEWMS with the addition of nanoparticles at different temperatures, some important parameters of NEWMS were calculated. As mentioned before, *G*_0_ is a practical plateau value of storage modulus. However, sometimes in actual experiments, it is hard to test this value or get it inaccurately. Researchers often use Equation (5) to calculate the plateau modulus *G′_∞_* [22]:

(5) $$G_{\infty }^{\prime }=2G{\prime \prime }_{\max}$$

Here, the modulus *G″*_max_ is the value of intersection point where *G′* is equal to *G″*. In addition, some important parameters of NEWMS are dependent on their structures, such as the mesh size *ξ_M_*, the entanglement length *l_e_*, the persistence length *l_p_*, and the contour length *L*. These parameters can be calculated from Equations (6)–(8) [22,29]:

(6) $$\frac{G_{\infty }^{\prime }}{G_{\min}^{\prime \prime }}\approx \frac{L}{l_{e}}$$

(7) $$l_{e}=\frac{\xi_{M}^{5/3}}{l_{p}^{2/3}}$$

(8) $$\xi_{M}={\left(\frac{k_{b}T}{G\prime_{\infty }}\right)}^{1/3}$$

Here, the modulus *G″*_min_ is the minimum of the loss modulus *G″* at high shear frequencies, as shown in Figure 3. The parameters *L*, *l_e_*, and *ξ_M_* decide the rheological properties of NEWMS. The value of *k_B_* is 1.38 × 10^−23^ J/K as the Boltzman constant and the persistence length *l_p_* is set to 15–25 nm according to previous studies [25,29]. According to the above information, these parameters of NEWMS are listed in Table 1.

### 3.2. Effects of Silica Nanoparticle Concentration

**Figure 5:**
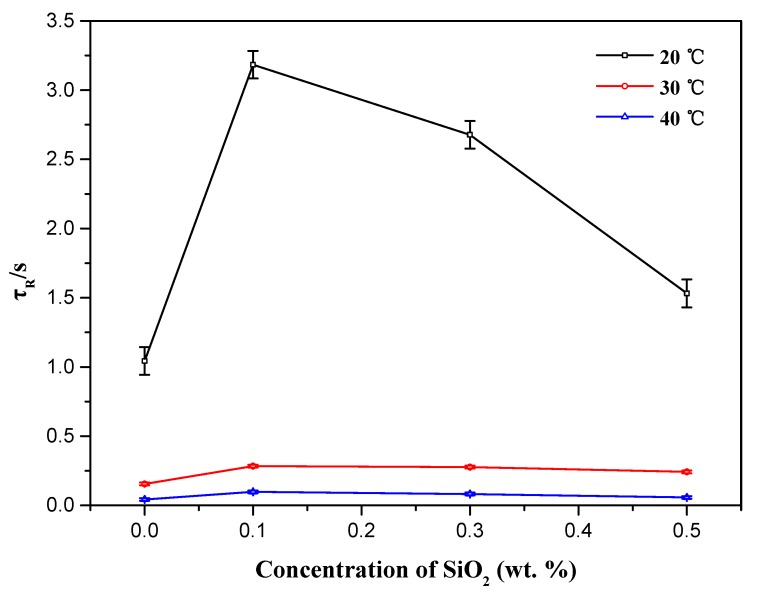
Relaxation time (*τ_R_*) of NEWMS with different nanoparticle concentrations at 20, 30, and 40 °C.

According to results listed in Table 1, at the same temperature, the addition of nanoparticles indeed improves the viscosities of wormlike micelles. With the further increase of silica nanoparticle concentrations, the viscosities of NEWMS reach the maximum and begin to decrease. In addition, the changes of relaxation time *τ_R_* and contour length *L* can be used to investigate the effect of nanoparticle concentration on NEWMS. As shown in Table 1, the contour length *L* is closely linked with the zero-shear viscosity *η*_0_*.* With the addition of nanoparticles, *L* gets increase, indicating that nanoparticles induce micellar growth. However, with the further addition, *L* decreases, suggesting that redundant nanoparticles destroy the original structure of wormlike micelles and shorten the micellar length. As shown in Figure 5, it can be observed that the relaxation time *τ_R_* is increased with the addition of silica nanoparticles, which has the same changing trend as that of the zero-shear viscosity *η*_0_*.* The plateau modulus *G′*_∞_, mesh size *ξ_M_* and entanglement length *l_e_* do not show variation distinctly, suggesting that the entangled network structure of NEWMS keeps integrated with the addition of nanoparticles. NEWMS with the addition of 0.1 wt % silica nanoparticles have the longest contour length *L*.

### 3.3. Effects of Temperature

In order to further investigate the rheological properties of NEWMS, the temperature effect is studied in the range of 20–40 °C and the corresponding parameters of NEWMS are listed in Table 1. At different temperatures, NEWMS show remarkable viscoelastic properties. With the increase of temperature, the viscosities of NEWMS begin to decrease dramatically.

**Figure 6:**
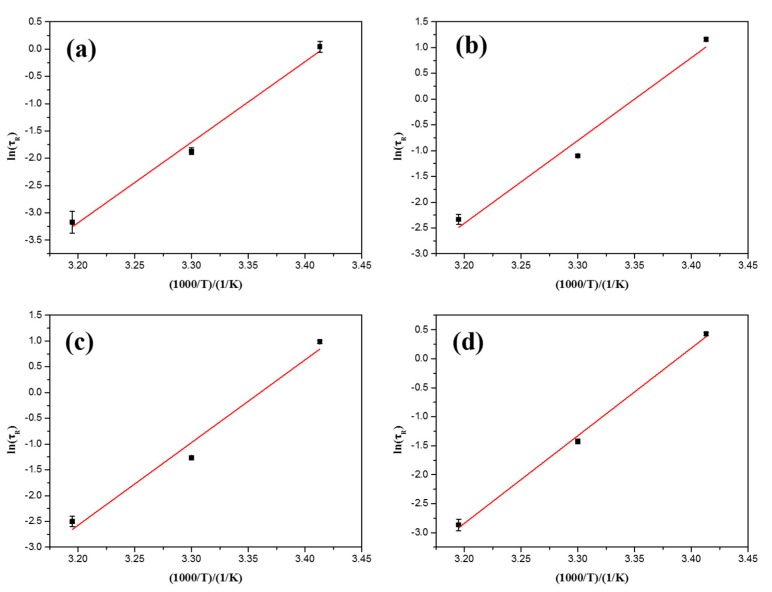
Arrhenius dependence of *τ_R_* with the reciprocal of absolute temperature for the NEWMS with different nanoparticle concentrations (**a**) 0 wt %; (**b**) 0.1 wt %; (**c**) 0.3 wt %; and (**d**) 0.5 wt %.

In addition, it can be observed that the contour length *L* decreases sharply with the increase of temperature, which results in the reduction of viscosities at higher temperature. This changing trend is consistent with the results of *τ_R_*, while the values of *l_e_* and *ξ_M_* keep nearly constant with the change of temperature, suggesting that the entangled network structure of NEWMS keeps integrated within the temperature range. The relationship between ln*τ_R_* and the reciprocal of the absolute temperature of NEWMS are plotted in Figure 6. The experimental data accords well to a linear relationship, indicating that the main relaxation time fits the Arrhenius relationships [22,29]:

(9) $$\tau_{R}=A\exp\left(\frac{E_{a}}{RT}\right)$$

Here, *E_a_* is the activation energy that describes the energy of individual micelles moving into an environment of surrounding micelles [23,30,31]. *R* is the gas constant and *A* is a constant. According to this Equation, *E_a_* values of NEWMS can be calculated and are listed in Table 2. By adding silica nanoparticles, *E_a_* values are larger than those of conventional wormlike micelles, indicating the influence of nanoparticles on micellar rheology.

### 3.4. Mechanism Discussion

According to previous work [3,15,20,21], there are many different thickening mechanisms of wormlike micelles with the addition of nanoparticles. Bandyopadhyay et al. proposed that the viscosity of wormlike micelles was increased because of additional electrostatic screening through contributions of silica nanoparticles to the bulk ion concentration [15]. Helgeson et al. found that the presence of nanoparticles does not significantly alter the electrostatic interactions between micelles [21]. They proposed that the addition of nanoparticles not only changes the surface electrical behavior of micellar molecules, but also forms a new kind of physical cross-link micellar structure, which can also be called a “double network”.

**Figure 7:**
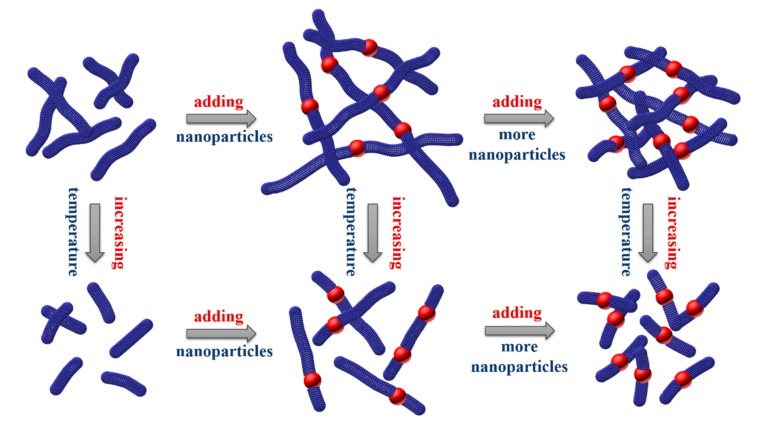
Illustration of the proposed mechanisms of micelle-particle junctions and effects of silica concentration and temperature on wormlike micelles.

In this work, the improvement of wormlike micelle rheological properties is obvious, and micellar solution with addition of 0.1 wt % silica shows the highest zero-shear viscosity. As for this phenomenon, the hydrophilic silica nanoparticles have negative charges with high surface area. Cationic CTAB surfactant molecules can adsorb on the surface of nanoparticles due to electrostatic attraction and hydrophilic interaction, forming a bilayer circular structure. With the addition of NaSal, the counter-ion can improve the aggregation of CTAB molecules. Meanwhile, bilayer circular structures would regroup and be involved in the formation of wormlike micelles, forming a new micelle-particle junction. This junction behaves as a bridging joint, improving micelles entangling with each other, causing the strength to increase and lead to the growth of micelles. With the increase in temperature, the contour length *L* of NEWMS begins to decrease sharply. At the same temperature, the contour length *L* and entanglement of NEWMS are larger than those of conventional wormlike micelles without nanoparticles. As can be seen in Figure 7, dilute silica concentrations can improve the aggregation of micelles and induce micellar growth. With the further increase of nanoparticle addition, excessive micelle-nanoparticle junctions make the network structure unconsolidated and weak. Redundant nanoparticles gather and aggregate due to surface energy. Such effects of aggregation improvement unexpectedly destroy the original network structure and form larger micellar molecular aggregates from overlapping and entanglement, which results in the decrease of viscosity.

## 4. Conclusions

In conclusion, a doubt about the effect of nanoparticles on the wormlike micelles has been clarified. With the addition of nanoparticles, viscosities of wormlike micelles cannot be continually increased. The viscosity of NEWMS can reach the maximum with the addition of nanoparticles. NEWMS have higher viscosity and better viscoelasticity than conventional wormlike micelles without silica nanoparticles. The added silica nanoparticles are attracted by hydrophilic headgroups of surfactant, forming a new micelle-particle junction. In addition, it can be observed that the viscosity of NEWMS is associated with values of *τ_R_* and *L*, indicating that nanoparticles lead to micellar growth and enhance bridging attractions between nanoparticles and micelles. However, with the further increase of nanoparticle concentration, the viscosity of NEWMS begins to decrease, which also reflects in values of *τ_R_* and *L*. Since the effects of aggregation improvement unexpectedly destroy the original network structure and form larger micellar molecular aggregates for overlapping and entanglement. Experimental results show that NEWMS with the addition of 0.1 wt % nanoparticles has the highest values of *η*_0_, *τ_R_*, and *L*. In addition, the temperature can cause a remarkable change for the contour length of NEWMS, while no effect on the entanglement length *l_e_* and mesh size *ξ_M_*. We expect this work can enrich the knowledge of NEWMS and widen their applications.

## Figures and Tables

**Table 1:** The rheological parameters of NEWMS with different mass fractions of silica nanoparticles at different temperatures.

*C_silica_* (wt %)	*T* (°C)	*η*_0_ (Pa·s)	*G′*_∞_ (Pa)	*G″*_min_ (Pa)	*τ_R_* (s)	*ξ_M_* (nm)	*l_e_* (nm)	*L* (nm)
0	20	15.311 ± 0.30	13.106 ± 0.02	1.118 ± 0.01	1.043 ± 0.10	67.62 ± 0.03	131–184	1536–2157
	30	2.242 ± 0.10	13.954 ± 0.02	2.978 ± 0.01	0.154 ± 0.01	66.91 ± 0.03	129–181	604–848
	40	0.694 ± 0.01	13.664 ± 0.02	5.415 ± 0.01	0.042 ± 0.01	68.13 ± 0.03	133–187	336–472
0.1	20	22.411 ± 0.30	14.691 ± 0.02	0.872 ± 0.01	3.184 ± 0.10	65.12 ± 0.03	123–173	2072–2914
	30	5.375 ± 0.10	14.634 ± 0.02	1.996 ± 0.01	0.276 ± 0.01	65.91 ± 0.03	126–177	924–1298
	40	1.484 ± 0.01	14.001 ± 0.02	3.021 ± 0.01	0.097 ± 0.01	67.64 ± 0.03	131–184	607–853
0.3	20	17.063 ± 0.30	14.139 ± 0.02	0.932 ± 0.01	2.677 ± 0.10	65.92 ± 0.03	126–177	1911–2685
	30	4.917 ± 0.10	13.382 ± 0.02	2.072 ± 0.01	0.283 ± 0.01	67.93 ± 0.03	132–186	853–1201
	40	1.113 ± 0.01	14.292 ± 0.02	3.289 ± 0.01	0.082 ± 0.01	67.11 ± 0.03	130–182	564–791
0.5	20	16.253 ± 0.30	11.537 ± 0.02	1.064 ± 0.01	1.532 ± 0.10	70.52 ± 0.03	140–197	1518–2136
	30	3.531 ± 0.10	12.633 ± 0.02	2.204 ± 0.01	0.241 ± 0.01	69.23 ± 0.03	136–192	779–1100
	40	0.943 ± 0.01	13.178 ± 0.02	4.101 ± 0.01	0.057 ± 0.01	68.92 ± 0.03	135–190	434–611

**Table 2:** The activation energy *E_a_* of NEWMS with different silica nanoparticle concentrations.

*C_silica_* (wt %)	*E_a_* (kJ/mol)
0	122
0.1	134
0.3	131
0.5	125
